# Hormone therapy and the decreased risk of dementia in women with depression: a population-based cohort study

**DOI:** 10.1186/s13195-022-01026-3

**Published:** 2022-06-16

**Authors:** Hyewon Kim, Juhwan Yoo, Kyungdo Han, Dong-Yun Lee, Maurizio Fava, David Mischoulon, Hong Jin Jeon

**Affiliations:** 1 Department of Psychiatry, Hanyang University Hospital, Seoul, South Korea; 2 Department of Biomedicine & Health Science, The Catholic University of Korea, Seoul, South Korea; 3 Department of Statistics and Actuarial Science, Soongsil University, Seoul, South Korea; 4 Department of Obstetrics and Gynecology, Samsung Medical Center, Sungkyunkwan University School of Medicine, Seoul, South Korea; 5 Depression Clinical and Research Program, Massachusetts General Hospital, Harvard Medical School, Boston, USA; 6 Department of Psychiatry, Depression Center, Samsung Medical Center, Sungkyunkwan University School of Medicine, Seoul, South Korea; 7 Department of Health Sciences & Technology, Department of Medical Device Management & Research, and Department of Clinical Research Design & Evaluation, Samsung Advanced Institute for Health Sciences & Technology (SAIHST), Sungkyunkwan University, Seoul, South Korea

**Keywords:** Hormone therapy, Hormone replacement therapy, Oral contraceptives, Depression, Alzheimer’s disease, Vascular dementia

## Abstract

**Background:**

The literature has shown depression to be associated with an increased risk of dementia. In addition, hormone therapy can be a responsive treatment option for a certain type of depression. In this study, we examined the association between hormone therapy, including lifetime oral contraceptive (OC) use, and hormone replacement therapy (HRT) after menopause with the occurrence of dementia among female patients with depression.

**Methods:**

The South Korean national claims data from January 1, 2005, to December 31, 2018, was used. Female subjects aged 40 years or older with depression were included in the analyses. Information on hormone therapy was identified from health examination data and followed up for the occurrence of dementia during the average follow-up period of 7.72 years.

**Results:**

Among 209,588 subjects, 23,555 were diagnosed with Alzheimer’s disease (AD) and 3023 with vascular dementia (VD). Lifetime OC usage was associated with a decreased risk of AD (OC use for < 1 year: HR, 0.92 [95% CI, 0.88–0.97]; OC use for ≥ 1 year: HR, 0.89 [95% CI, 0.84–0.94]), and HRT after menopause was associated with a decreased risk of AD (HRT for < 2 years: HR, 0.84 [95% CI, 0.79–0.89]; HRT for 2–5 years: HR, 0.80 [95% CI, 0.74–0.88]; and HRT for ≥ 5 years : HR, 0.78 [95% CI, 0.71–0.85]) and VD (HRT < 2 years: HR, 0.82 [95% CI, 0.71–0.96]; HRT for 2–5 years: HR, 0.81 [95% CI, 0.64–1.02]; and HRT for ≥ 5 years: HR, 0.61 [95% CI, 0.47–0.79]).

**Conclusions:**

In this nationwide cohort study, lifetime OC use was associated with a decreased risk of AD, and HRT after menopause was associated with a decreased risk of AD and VD among female patients with depression. However, further studies are needed to establish causality.

**Supplementary Information:**

The online version contains supplementary material available at 10.1186/s13195-022-01026-3.

## Background

Depression is a common psychiatric disorder associated with significant morbidity [1]. Although the mechanism underlying depression is yet to be established, one potential etiology is a change in the levels of female sex hormones. Epidemiological studies have shown that women have about twice the risk of depression as men [2–4], and perimenopausal or postmenopausal women who experience a rapid decline in levels of female sex hormones have an increased risk of depression compared to premenopausal women [5]. A placebo-controlled study showed an increase in the depressive symptoms among premenopausal women who were rendered temporally hypogonadal by gonadotropin-releasing hormone agonists [6]. In addition, women can experience reproductive depression such as premenopausal dysphoric disorder and postpartum depression when rapid changes in the levels of estrogen occur. On the other hand, female sex hormones may help prevent and improve depressive symptoms; a cross-sectional study showed that women, aged ≥ 60 years, who received hormone replacement therapy (HRT) had lower rates of depressive symptoms than those not taking HRT [7]. Randomized controlled studies have likewise shown the effect of hormone therapy on perimenopausal women experiencing depressive disorders [8, 9]. In particular, hormonal therapy has been proven as an effective treatment option for reproductive depression [10].

Previous studies have suggested that depression is associated with an increased risk of dementia [11–13]. The Women’s Health Initiative Memory Study showed that baseline depressive disorder was associated with an increased risk of incident mild cognitive impairment (HR, 1.98) and probable dementia (HR, 2.03) among postmenopausal women without cognitive impairment aged 65 to 79 [14]. Similarly, prospective studies revealed that late-life depression is linked to dementia with a 2- to 5-fold increased risk [15–20]. Although fewer studies have focused on early-onset depression compared to late-life depression, early onset of depression was also associated with a 2- to 4-fold increased risk of dementia [21–24]. Depending on the type of dementia, depression was associated with a 1.2- to 4.6-fold increased risk of Alzheimer’s disease (AD) [13, 15, 21, 23, 25–27], and a 1.2 to 2.4-fold increased risk of vascular dementia (VD) [20, 25]. Overall, a recent meta-analysis study found that depression is one of the most significant risk factors for dementia, with a relative risk of 1.99 [28]. However, in these cases, the increased risk is not always the cause. Depression, for example, can be an actual risk factor or an early manifestation of dementia, and the underlying mechanism is yet unknown. Alternatively, it might be a case of pseudodementia, in which cognitive impairment caused by depression is misdiagnosed as dementia.

Previous studies have shown inconsistent results on the association between hormone therapy and dementia. In a longitudinal study, the risk of incident AD was reduced by about half in hormone replacement users compared to nonusers [29]. Hormone therapy, on the other hand, showed no significant effect on the prevention of dementia or the enhancement of cognition in elderly women who had been in menopause for several years. A randomized controlled trial showed that estrogen plus progestin therapy among postmenopausal women aged 65 years or older doubled the risk of probable dementia compared to placebo, and no protective effect on mild cognitive impairment was found [30]. In the Women’s Health Initiative Memory Study, estrogen plus progestin hormone therapy had a negative impact on verbal memory, but a positive impact on figural memory (committing objects to visual memory and then recognizing them when shown in a stream of different objects) was observed [31]. Additionally, there was no preventive effect of dementia on the estrogen-alone group [32]. When compared to placebo, 20 weeks of unopposed estradiol replacement therapy did not improve cognitive performance in women aged 70 or older [33].

The idea that estrogen has neuroprotective properties is a relatively new one [34, 35]. This research suggests that estrogen may affect both neurodegenerative disorders such as dementia and affective disorders such as depression. However, the association between depression, dementia, and estrogen is poorly understood, and there is still no evidence on the effect of hormone therapy on dementia among women with depression.

In this study, we used a national cohort of South Korea to examine the association between hormone therapy including oral contraceptives (OCs) and HRT with the risk of dementia in postmenopausal women. We hypothesized that (1) lifetime OC use is associated with the risk of dementia among patients with depression and (2) postmenopausal HRT is associated with the risk of dementia among patients with depression.

## Methods

### Data source

The database from the National Health Insurance Sharing Service (NHISS) of the National Health Insurance Service (NHIS) of South Korea was used [36, 37]. The NHIS is a public institution responsible for operating mandatory universal health insurance, and nearly 97% of the South Korean population is enrolled in this service, while the remaining 3% is covered by the Medical Aid Program. The NHISS contains medical service claims data such as admissions, emergency room visits, ambulatory care visits, and pharmaceutical services.

The database from the National Cancer Screening Program (NCSP) was also used [38]. The NCSP contains screening data for stomach, liver, colorectal, breast, and cervical cancers, and each screening examination was conducted based on the age of the participants. All South Korean women aged 40 years or older are encouraged to be screened for breast and cervical cancer biennially. Even though the screening program was voluntary, participation rates reached up to 70% [39].

The data of NHISS and NCSP were anonymized by using individual research numbers instead of social security numbers to protect the privacy of the individuals. The study protocol was approved by the Institutional Review Board of the Samsung Medical Center (IRB No. 2021-03-108).

### Case identification

**Fig. 1:**
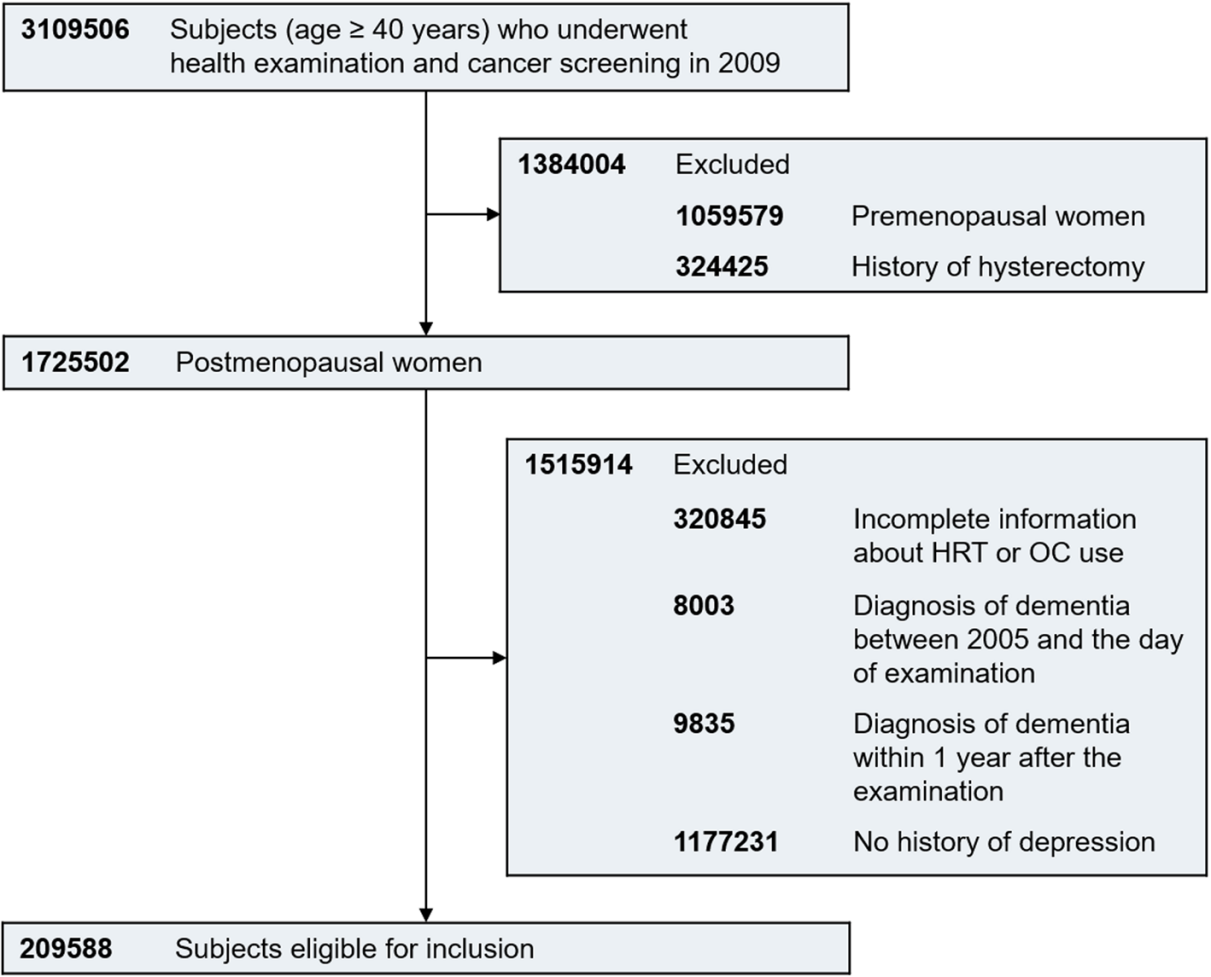
Flowchart

Among 3,109,506 female subjects aged 40 years or older who underwent health examination and screening for breast/cervical cancer on the same day from January 1, 2009, to December 31, 2009, 1,725,502 were identified as postmenopausal without a history of hysterectomy. Those with incomplete information about HRT or OC use (*n* = 320,845) and those with a previous diagnosis of dementia before the examination (*n* = 8003) were excluded to secure the first diagnosis of dementia. In addition, 9835 subjects who were diagnosed with dementia within 1 year after the examination were excluded to eliminate the effect of a temporary increase in the diagnosis of dementia by detecting it at the time of examination. After excluding 1,177,231 subjects with no history of depression, 209,588 subjects were deemed eligible for our study, and their medical records were followed until December 31, 2018 (Fig. 1). The diagnosis of depression (F32 and F33) was defined based on the International Statistical Classification of Disease and Related Health Problems 10th revision (ICD-10).

### Lifetime uses of oral contraceptives

Information on the usage of OC was extracted from self-administered questionnaire data from the cancer screening program. The question was “Are you on or have you ever taken oral contraceptive pills?” Subjects were asked to choose a response among “never,” “use for less than 1 year,” “use for more than 1 year,” or “unknown.”

### Hormone replacement therapy after menopause

Information on HRT was extracted from the self-administered questionnaire data from a cancer screening program. The question was “Are you on or have you ever taken hormonal agents to relieve postmenopausal symptoms?” Subjects were asked to choose a response among “never,” “use for less than 2 years,” “use of 2–5 years,” “use for more than 5 years,” or “unknown.”

### Outcomes

The main outcome was the diagnosis of dementia (F00 and F30 for AD; F01 for VD; and F02, F03, and F31 for other dementia) and the prescription of one or more medications for dementia during the follow-up period. When a subject had more than one code for dementia diagnosis, the subject was classified based on the principal diagnosis. If both AD and VD codes were included in the additional diagnosis of a subject, the subject was classified based on the principal diagnosis of the next hospital visit, and if both AD and VD codes remained as additional diagnosis codes, the subject was classified as another dementia group. Prescribed medications for dementia included donepezil, rivastigmine, galantamine, or memantine.

### Covariates

The body mass index (BMI) was calculated using the subjects’ weight and height measured on the day of the cancer screening examination. Lifestyle factors such as smoking, alcohol consumption, and exercise were identified from the NCSP self-questionnaire. Regular exercise was defined as performing a moderate physical activity for more than 30 min at least five times a week or vigorous physical activity for more than 20 min at least three times a week. Subjects were categorized into levels of income based on the payment of health insurance. Comorbid physical illnesses including hypertension, diabetes mellitus, and dyslipidemia were identified based on ICD-10 codes from past medical records.

### Statistical analyses

Continuous variables were displayed as mean ± standard deviation (SD), while categorical variables were displayed as number and percentage. The Student *t*-test was used to compare the differences in individual factors between the groups. Cox proportional hazards regression analyses were conducted to identify the association between hormone therapy and the diagnosis of dementia, and censored for the occurrence of dementia or death. The proportional hazard assumption was visually tested using the Schoenfeld residuals plot and the log-log survival plot. The hazard ratios (HRs) and corresponding 95% confidence intervals (CIs) revealed the magnitude of risk of dementia based on the duration of hormone therapy. Statistical analyses were performed using SAS version 9.4 (SAS Institute Inc., Cary, NC, USA).

## Results

Table 1 shows the baseline characteristics of the subjects. The mean age of the subjects was 61.54 years (SD, 7.83) in the non-dementia group and 70.46 years (SD, 6.59) in the dementia group (*p* < 0.0001). In comparison with the non-dementia group, the dementia group had a lower proportion of alcohol consumption and regular exercise; a greater prevalence of hypertension, diabetes mellitus, and dyslipidemia; and lower usage of OC and experience with HRT. Table 2 shows the baseline characteristics of subjects according to the duration of hormone therapy. Regarding the use of OCs, longer OC users were more likely to have a higher BMI, lower total cholesterol and LDL levels, currently smoke, drink heavily, have the highest income level, have dyslipidemia, and experience menopause later in life. Regarding HRT, longer HRT users were more likely to exercise regularly; have lower diastolic blood pressure, fasting glucose, and total cholesterol; and have less dyslipidemia.

**Table 1:** Baseline characteristics of study subjects

	Non-dementia group (***n*** = 179,723)	Dementia group (***n*** = 29,865)	***P***
Age (years)	61.54 ± 7.83	70.46 ± 6.59	< 0.0001
BMI (kg/m^2^)	24.26 ± 3.14	24.19 ± 3.31	< 0.001
Systolic BP (mmHg)	125.01 ± 15.81	128.73 ± 16.23	< 0.0001
Diastolic BP (mmHg)	76.63 ± 9.98	77.77 ± 10.08	< 0.0001
Fasting glucose (mg/dL)	99.89 ± 24.24	104.63 ± 30.56	< 0.0001
Total cholesterol (mg/dL)	206.62 ± 46.60	205.47 ± 42.25	< 0.0001
HDL (mg/dL)	57.50 ± 35.39	56.05 ± 35.84	< 0.0001
LDL (mg/dL)	125.28 ± 77.35	123.31 ± 81.84	< 0.0001
Smoking status			0.208
Never	172,482 (95.97)	28,606 (95.78)	
Ex-smoker	2121 (1.18)	353 (1.18)	
Current smoker	5120 (2.85)	906 (3.03)	
Alcohol consumption^a^			< 0.0001
None	160,398 (89.25)	28,075 (94.01)	
Mild	18,428 (10.25)	1719 (5.76)	
Heavy	897 (0.50)	71 (0.24)	
Levels of income			< 0.0001
Medical aid + 1st quartile (the lowest)	37,606 (20.92)	5645 (18.9)	
2nd quartile	31,760 (17.67)	4842 (16.21)	
3rd quartile	46,044 (25.62)	6739 (22.56)	
4th quartile (the highest)	64,313 (35.78)	12,639 (42.32)	
Regular exercise	34,103 (18.98)	3792 (12.70)	< 0.0001
Hypertension	90,215 (50.20)	20,248 (67.80)	< 0.0001
Diabetes mellitus	26,506 (14.75)	7369 (24.67)	< 0.0001
Dyslipidemia	70,467 (39.21)	12,847 (43.02)	< 0.0001
Age at menarche (years)	16.50 ± 1.82	16.92 ± 1.79	< 0.0001
Age at menopause (years)	49.99 ± 4.07	49.27 ± 4.56	< 0.0001
Duration of fertility (years)	33.49 ± 4.45	32.35 ± 4.95	< 0.0001
Duration of OC use (years)			< 0.0001
Never	149,286 (83.06)	26,008 (87.09)	
< 1	17,771 (9.89)	2156 (7.22)	
≥ 1	12,666 (7.05)	1701 (5.70)	
Duration of HRT (years)			< 0.0001
Never	140,749 (78.31)	26,781 (89.67)	
< 2	22,097 (12.30)	1735 (5.81)	
2–5	9182 (5.11)	663 (2.22)	
≥ 5	7695 (4.28)	686 (2.30)	

Data are expressed as the mean ± standard deviation, SD, or *n* (%)

*Abbreviations*: *BMI* body mass index, *BP* blood pressure, *HDL* high-density lipoprotein, *LDL* low-density lipoprotein, *OC* oral contraceptives, *HRT* hormone replacement therapy

^a^Alcohol consumption: mild = up to 30 g (equivalent to 3 drinks) a day; heavy = more than 30 g a day

**Table 2:** Baseline characteristics of study subjects according to the duration of hormone therapy

	Duration of OC use (years)				Duration of HRT (years)				
	Never (***n*** = 175,294)	< 1 (***n*** = 19,927)	≥ 1 (***n*** = 14,367)	***P***	Never (***n*** = 167,530)	< 2 (***n*** = 23,832)	2–5 (***n*** = 9845)	≥ 5 (***n*** = 8381)	***P***
Age (years)	62.96 ± 8.45	61.59 ± 7.37	62.70 ± 7.12	< 0.0001	63.62 ± 8.45	59.10 ± 6.87	59.35 ± 6.36	61.43 ± 60	< 0.0001
BMI (kg/m^2^)	24.21 ± 3.18	24.35 ± 3.08	24.61 ± 3.12	< 0.001	24.33 ± 3.22	24.00 ± 2.96	23.78 ± 2.86	23.85 ± 2.79	< 0.0001
Systolic BP (mmHg)	125.55 ± 15.98	125.06 ± 15.71	126.15 ± 15.62	< 0.0001	126.30 ± 16.04	122.65 ± 15.08	122.07 ± 15.01	122.77 ± 15.19	< 0.0001
Diastolic BP (mmHg)	76.82 ± 10.01	76.55 ± 9.99	76.82 ± 9.95	0.0019	77.14 ± 10.04	75.59 ± 9.80	75.20 ± 9.74	75.04 ± 9.68	< 0.0001
Fasting glucose (mg/dL)	100.52 ± 25.38	100.40 ± 24.65	101.41 ± 25.07	0.0002	101.29 ± 26.27	98.14 ± 20.76	97.26 ± 20.47	96.81 ± 20.59	< 0.0001
Total cholesterol (mg/dL)	206.66 ± 46.53	205.79 ± 41.49	204.82 ± 45.48	< 0.0001	207.16 ± 46.96	205.86 ± 44.10	202.37 ± 38.71	198.73 ± 38.30	< 0.0001
HDL (mg/dL)	57.38 ± 36.31	56.87 ± 30.07	56.86 ± 31.51	0.0468	57.30 ± 36.50	57.68 ± 32.82	57.26 ± 28.92	56.09 ± 27.50	0.0059
LDL (mg/dL)	125.19 ± 80.79	124.31 ± 60.11	123.64 ± 64.17	0.0309	125.16 ± 74.84	125.18 ± 60.46	123.93 ± 113.57	122.51 ± 121.11	0.0105
Smoking status				0.208					< 0.0001
Never	168,431 (96.08)	19,019 (95.44)	13,638 (94.93)		161,079 (96.15)	22,713 (95.30)	9334 (94.81)	7962 (95.00)	
Ex-smoker	1901 (1.08)	320 (1.61)	253 (1.76)		1773 (1.06)	371 (1.56)	177 (1.80)	153 (1.83)	
Current smoker	4962 (2.83)	588 (2.95)	476 (3.31)		4678 (2.79)	748 (3.14)	334 (3.39)	266 (3.17)	
Alcohol consumption^a^				< 0.0001					< 0.0001
None	158,564 (90.46)	17,410 (87.37)	12,499 (87.00)		152,177 (90.84)	20,603 (86.45)	8447 (85.80)	7246 (86.46)	
Mild	15,998 (9.13)	2390 (11.99)	1759 (12.24)		14,633 (8.73)	3085 (12.94)	1337 (13.58)	1092 (13.03)	
Heavy	732 (0.42)	127 (0.64)	109 (0.76)		720 (0.43)	144 (0.60)	61 (0.62)	43 (0.51)	
Levels of income				0.0034					< 0.0001
Medical aid + 1st quartile (the lowest)	36,339 (20.73)	4079 (20.47)	2833 (19.72)		34,427 (20.55)	5190 (21.78)	2022 (20.54)	1612 (19.23)	
2nd quartile	30,713 (17.52)	3454 (17.33)	2435 (16.95)		29,337 (17.51)	4276 (17.94)	1697 (17.24)	1292 (15.42)	
3rd quartile	44,113 (25.17)	5060 (25.39)	3610 (25.13)		42,234 (25.21)	5957 (25.00)	2503 (25.42)	2089 (24.93)	
4th quartile (the highest)	64,129 (36.58)	7334 (36.80)	5489 (38.21)		61,532 (36.73)	8409 (35.28)	3623 (36.80)	3388 (40.42)	
Regular exercise	30,862 (17.61)	4005 (20.10)	3028 (21.08)	< 0.0001	28,011 (16.72)	5330 (22.36)	2372 (24.09)	2182 (26.04)	< 0.0001
Hypertension	91,855 (52.40)	10,346 (51.92)	8262 (57.51)	< 0.0001	91,657 (54.71)	10,477 (43.96)	4322 (43.90)	4007 (47.81)	
Diabetes mellitus	28,077 (16.02)	3155 (15.83)	2643 (18.40)	< 0.0001	28,809 (17.20)	2891 (12.13)	1121 (11.39)	1054 (12.58)	
Dyslipidemia	69,087 (39.41)	8101 (40.65)	6126 (42.64)	< 0.0001	67,068 (40.03)	9434 (39.59)	3712 (37.70)	3100 (36.99)	
Age at menarche (years)	16.57 ± 1.83	16.49 ± 1.83	16.56 ± 1.81	< 0.0001	16.63 ± 1.81	16.30 ± 1.86	16.25 ± 1.82	16.35 ± 1.84	
Age at menopause (years)	49.83 ± 4.15	50.13 ± 4.15	50.24 ± 4.19	< 0.0001	49.88 ± 4.14	50.07 ± 4.00	50.04 ± 4.18	49.41 ± 4.78	
Duration of fertility (years)	33.26 ± 4.54	33.65 ± 4.52	33.68 ± 4.54	< 0.0001	33.25 ± 4.55	33.78 ± 4.33	33.79 ± 4.43	33.06 ± 4.98	
Duration of OC use (years)									< 0.0001
Never					144,214 (86.08)	17,651 (74.06)	7295 (74.1)	6134 (73.19)	
< 1					13,738 (8.20)	3817 (16.02)	1301 (13.21)	1071 (12.78)	
≥ 1					9578 (5.72)	2364 (9.92)	1249 (12.69)	1176 (14.03)	
Duration of HRT (years)				< 0.0001					
Never	144,214 (82.27)	13,738 (68.94)	9578 (66.67)						
< 2	17,651 (10.07)	3817 (19.15)	2364 (16.45)						
2–5	7295 (4.16)	1301 (6.53)	1249 (8.69)						
≥ 5	6134 (3.50)	1071 (5.37)	1176 (8.19)						

Data are expressed as the mean ± standard deviation, SD, or *n* (%)

*Abbreviations*: *BMI* body mass index, *BP* blood pressure, *HDL* high-density lipoprotein, *LDL* low-density lipoprotein, *OC* oral contraceptives, *HRT* hormone replacement therapy

^a^Alcohol consumption: mild = up to 30 g (equivalent to 3 drinks) a day; heavy = more than 30 g a day

The average follow-up length was 7.72 years (SD, 1.87). Overall, 29,865 patients were newly diagnosed with dementia during the follow-up period, and the incidence rate was 18.47 per 1000 person-years. Among them, 23,555 were classified as AD subgroup with an incidence rate of 14.57 per 1000 person-years, and 3023 were classified as VD subgroup with an incidence rate of 1.87 per 1000 person-years.

In the adjusted model, compared to those who had never used OC, those who had used OC showed a decreased risk of all dementia (OC use for < 1 year: HR, 0.92 [95% CI, 0.88–0.96]; OC use for ≥ 1 year: HR, 0.90 [95% CI, 0.86–0.95]). Although OC use was associated with a decreased risk of AD (OC use for < 1 year: HR, 0.92 [95% CI, 0.88–0.97]; OC use for ≥ 1 year: HR, 0.89 [95% CI, 0.84–0.94]), there was no significant association between OC use and VD.

**Fig. 2:**
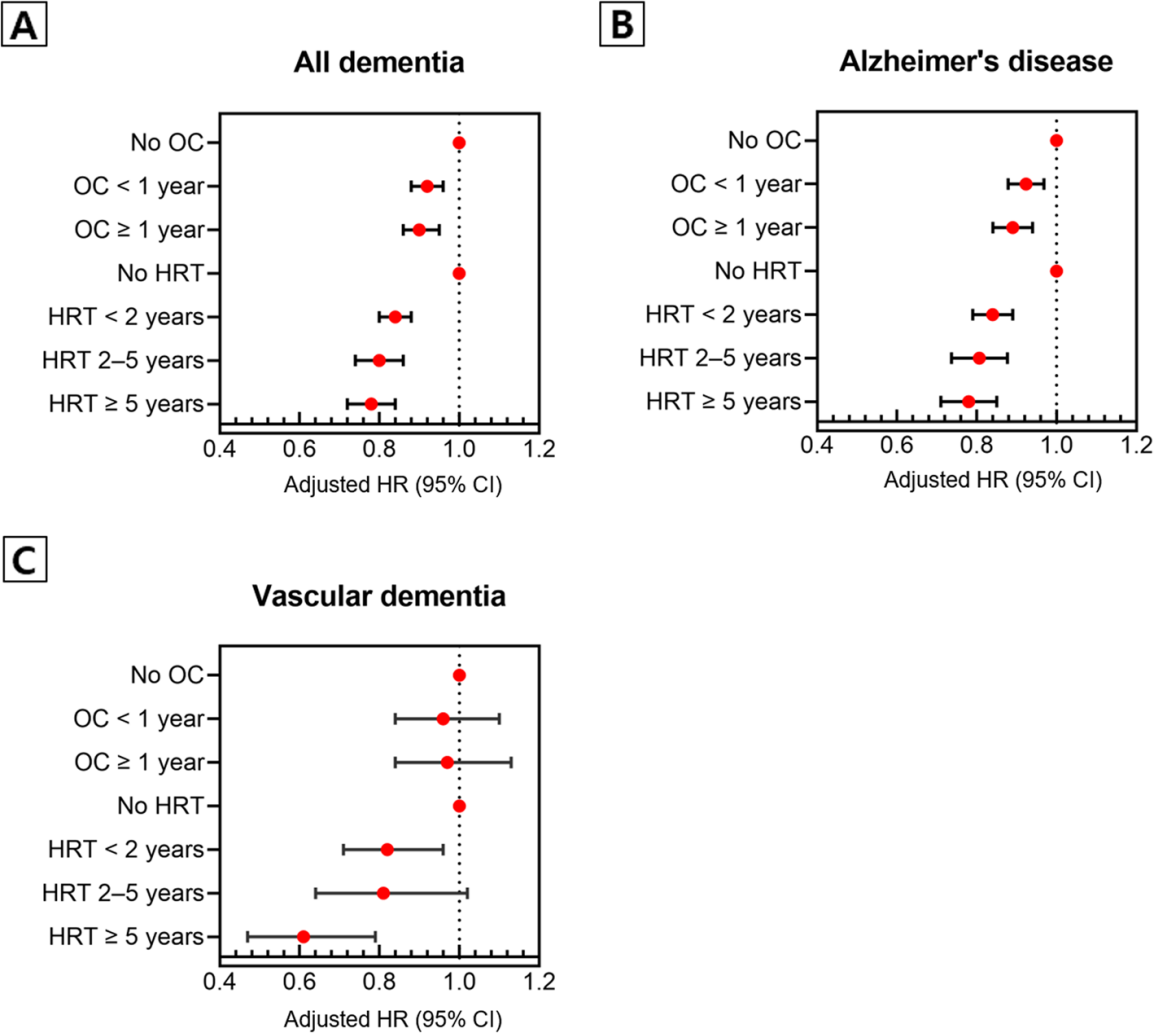
Forest plots showing the hazard ratios and corresponding 95% confidence intervals of hormone therapy on the diagnosis of dementia. OC, oral contraceptives; HRT, hormone replacement therapy

In the adjusted model, compared to those who had never received HRT, subjects who had received HRT showed a decreased risk of all dementia (HRT for < 2 years: HR, 0.84 [95% CI, 0.80–0.88]; HRT for 2–5 years: HR, 0.80 [95% CI 0.74–0.86]; and HRT for ≥ 5 years: HR, 0.78 [95% CI, 0.72–0.84]). The HRT was associated with a decreased risk of AD (HRT for < 2 years: HR, 0.84 [95% CI, 0.79–0.89]; HRT for 2–5 years: HR, 0.80 [95% CI, 0.74–0.88]; and HRT for ≥ 5 years : HR, 0.78 [95% CI, 0.71–0.85]) and VD (HRT < 2 years: HR, 0.82 [95% CI, 0.71–0.96]; HRT for 2–5 years: HR, 0.81 [95% CI, 0.64–1.02]; and HRT for ≥ 5 years: HR, 0.61 [95% CI, 0.47–0.79]) (Table 3 and Fig. 2).

**Table 3:** Hazard ratios and 95% confidence intervals of the duration of hormone therapy on the diagnosis of dementia

	All dementia		Alzheimer’s disease		Vascular dementia	
	Crude	Adjusted^**a**^	Crude	Adjusted^**a**^	Crude	Adjusted^**a**^
	Hazard ratio (95% confidence interval)					
Duration of OC use (years)						
Never	1 (ref.)	1 (ref.)	1 (ref.)	1 (ref.)	1 (ref.)	1 (ref.)
< 1	0.71 (0.68, 0.74)	0.92 (0.88, 0.96)	0.71 (0.67, 0.74)	0.92 (0.88, 0.97)	0.76 (0.66, 0.87)	0.96 (0.84, 1.10)
≥ 1	0.78 (0.74, 0.82)	0.90 (0.86, 0.95)	0.77 (0.73, 0.81)	0.89 (0.84, 0.94)	0.87 (0.75, 1.01)	0.97 (0.84, 1.13)
Duration of HRT (years)						
Never	1 (ref.)	1 (ref.)	1 (ref.)	1 (ref.)	1 (ref.)	1 (ref.)
< 2	0.43 (0.41, 0.45)	0.84 (0.80, 0.88)	0.43 (0.40, 0.45)	0.84 (0.79, 0.89)	0.44 (0.38, 0.51)	0.82 (0.71, 0.96)
2–5	0.40 (0.37, 0.43)	0.80 (0.74, 0.86)	0.39 (0.36, 0.43)	0.80 (0.74, 0.88)	0.42 (0.33, 0.53)	0.81 (0.64, 1.02)
≥ 5	0.48 (0.45, 0.52)	0.78 (0.72, 0.84)	0.48 (0.44, 0.52)	0.78 (0.71, 0.85)	0.39 (0.30, 0.51)	0.61 (0.47, 0.79)

*Abbreviations*: *OC* oral contraceptives, *HRT* hormone replacement therapy

^a^Adjusted for age, body mass index, level of income, current smoking, drinking status, regular exercise, diabetes mellitus, hypertension, and dyslipidemia

Supplementary Table 1 shows the results of Cox proportional hazards regression analyses with age as the time scale instead of risk time. As the result, the reduction in the risk of dementia according to the use of OCs and HRT was attenuated compared to that with risk time as the time-scale in the crude model, but after adjusting covariables, the magnitude of risk reduction was similar to that of main results.

Supplementary Table 2 presents the results of sensitivity analyses, which include those who were excluded from the main analyses because they had answered “unknown” to the question about the use of OC or HRT. Their associations with the risk of dementia were not statistically significant compared to those who had never used OC or HRT, respectively.

Supplementary Table 3 shows the results of sensitivity analyses excluding those who were diagnosed with dementia 3 years after baseline, and the patterns of results were similar to those of the main analyses.

Supplementary Table 4 shows the results of the regression model adding age at menopause as an adjusting variable in the main model, and the results were similar to those of the main analyses.

## Discussion

This study showed an association between hormone therapy and the risk of dementia among female patients with depression. In this study, three major findings were presented. First, lifetime use of OCs was associated with a decreased risk of AD. Second, HRT was associated with a decreased risk of AD and VD. Third, the risk of dementia decreased with the increased duration of hormone therapy.

Our results showed that HRT prescribed to relieve postmenopausal symptoms was associated with a lower risk of dementia than lifetime OC use. Postmenopausal symptoms including vasomotor symptoms are prevalent across women’s late menopausal transition stage and early postmenopausal stage [40]. Considering the mean age of menopause of subjects was about 50 years, the users of HRT might have experienced menopausal symptoms in their late 40s or early 50s. The Women’s Health Initiative Memory Study, which reported that hormone therapy had no preventive effect or increased risk for dementia, targeted women over 65 years of age [30, 32]. The Baltimore Aging Study reported the preventive effect of estrogen therapy on AD in postmenopausal women in a longitudinal follow-up, and the study included community-dwelling adult women who were not restricted to a specific age group [29]. Overall, these findings suggest that hormone therapy conducted at a “critical period” in reproductive aging may reduce the risk of dementia in late life.

When comparing the results of a study that used the same database as our study but targeted the general population rather than patients with depression [41], the preventive effect of lifetime OC use on AD and VD was similar in the general population and depression patients. However, regarding HRT after menopause, there was a risk reduction of 13–19% in AD and 14–23% in VD among the general population, and our study showed a higher risk reduction both in AD and VD. This result suggests that HRT after menopause lowers the risk of dementia in patients with depression more than in the general population. Moreover, the reduced risk of VD in those who received HRT for more than 5 years was found to be more robust: 39% in patients with depression compared to 22% in the general population.

Our findings have implications for recognizing the preventive effect of hormone therapy on the occurrence of dementia in patients with depression, but several concerns need to be addressed in terms of causality. In a selected population of postmenopausal women with depression, a significant association between hormone therapy and dementia with a dose-response relationship suggests that hormone therapy could be a meaningful preventive factor for dementia. However, despite the fact that hormone therapy preceded the diagnosis of dementia, it is difficult to believe that it fits the temporality of evidence required to show a causal association between them, particularly in AD, because the initial pathologic change of AD, the accumulation of amyloid-β, is generally known to precede symptoms by 10–15 years [42]. Although the results were still significant after eliminating the occurrence of dementia up to 3 years after the diagnosis of depression in the sensitivity analyses, given the much longer preclinical time, a reversed causality of depression with early presentation of dementia cannot be ruled out.

Although the nature of the association between depression and dementia is unclear, potential biological mechanisms include vascular changes, alterations in glucocorticoid steroids, hippocampal atrophy, deposition of β-amyloid plaques, inflammatory changes, and deficits in neurotrophin nerve growth factors [43]. In addition to the direct neuroprotective actions of estrogen [44–46], the prominent HRT-associated reduction in risk of dementia in depression patients compared to the general population suggests that hormone therapy may affect this pathway linking depression to dementia. Previous studies suggest that hormones may affect the occurrence of dementia in patients with depression via these mechanisms: in a female rodent study, decreasing the level of estrogen by ovariectomy increased amyloid-β oligomers, while estrogen replacement in AD model mice decreased amyloid-β oligomers [47]; in a clinical study, patients with AD had a lower level of estradiol in the cerebrospinal fluid than controls, and the level of estradiol was inversely correlated with β-amyloid concentration, suggesting beneficial effects of HRT on the development and course of AD [48]; estrogen has been shown to act on glial cells to maintain neurovascular function and regulate neuroinflammation [49, 50]; and loss of female sex hormone exacerbated impaired cerebral blood flow and cognitive function via heightened vasoconstriction, reduced vasodilation, and impaired nitric oxide signaling [51].

To the best of our best knowledge, this is the first study to longitudinally follow the effect of hormone therapy on dementia among patients with depression. Our findings present real-world evidence that hormone therapy can be beneficial in the prevention of dementia in patients with depression.

## Limitations

This study has several limitations. First, because the data on lifetime OC usage and HRT after menopause were identified retrospectively from responses on the self-administered questionnaires, there may be recall bias. Second, we could not verify information on hormone treatment formulation and dosage and the timing of therapy. Although the question for HRT included the phrase “to relieve postmenopausal symptoms,” subjects could perceive postmenopausal symptoms subjectively, and there was no information on when to start HRT after menopause. Third, we could not include several major risk factors of dementia in the analyses such as the ε4 allele of the Apolipoprotein E (APOE), hearing loss, history of traumatic brain injury, and the level of education [52]. Instead of the level of education, the level of income was included in the analyses as the level of education is a major contributor to income [53].

## Conclusions

Among women with depression, lifetime OC use was associated with a decreased risk of AD, and HRT after menopause was associated with a decreased risk of AD and VD. In addition, as the duration of hormone therapy increased, the risk of dementia decreased. Our findings suggest that hormone therapy among patients with depression may be beneficial in the prevention of dementia, and this could represent valuable evidence for physicians’ clinical decision-making. Further prospective studies are needed to secure stronger causality and safety information.

## Supplementary Information

**Additional file 1: Supplementary Table 1.** Hazard ratios and 95% confidence intervals of the duration of hormone therapy on the diagnosis of dementia with age as the time-scale. **Supplementary Table 2.** Hazard ratios and 95% confidence intervals of the duration of hormone therapy on the diagnosis of dementia including those who responded as “unknown” to the questions about hormone therapy. **Supplementary Table 3.** Hazard ratios and 95% confidence intervals of the duration of hormone therapy on the diagnosis of dementia excluding those who were diagnosed with dementia 3 years after the diagnosis of depression. **Supplementary Table 4.** Hazard ratios and 95% confidence intervals of the duration of hormone therapy on the diagnosis of dementia adding age at menopause as an adjusting variable.

## Data Availability

Publicly available datasets were analyzed in this study. This data can be found in the following: https://nhiss.nhis.or.kr/.
