# Supplementary material for: Hormone therapy and the decreased risk of dementia in women with depression: a population-based cohort study
Source: Alzheimers Res Ther. 2022 Jun 16;14:83. doi: 10.1186/s13195-022-01026-3 (PMC9202170; doi:10.1186/s13195-022-01026-3)
Supplement: Supplementary file 1 — Additional file 1: Supplementary Table 1. Hazard ratios and 95% confidence intervals of the duration of hormone therapy on the diagnosis of dementia with age as the time-scale. Supplementary Table 2. Hazard ratios and 95% confidence intervals of the duration of hormone therapy on the diagnosis of dementia including those who responded as “unknown” to the questions about hormone therapy. Supplementary Table 3. Hazard ratios and 95% confidence intervals of the duration of hormone therapy on the diagnosis of dementia excluding those who were diagnosed with dementia 3 years after the diagnosis of depression. Supplementary Table 4. Hazard ratios and 95% confidence intervals of the duration of hormone therapy on the diagnosis of dementia adding age at menopause as an adjusting variable. [file 13195_2022_1026_MOESM1_ESM.docx]

**Supplementary Table 1. Hazard ratios and 95% confidence intervals of the duration of hormone therapy on the diagnosis of dementia with age as the time-scale.**

|  |  | **All dementia** | | **Alzheimer’s disease** | | **Vascular dementia** | |
| --- | --- | --- | --- | --- | --- | --- | --- |
|  |  | **Crude** | **Adjusted^*^** | **Crude** | **Adjusted^*^** | **Crude** | **Adjusted^*^** |
|  |  | **Hazard ratio (95% Confidence interval)** | | | | | |
| Duration of OC use (years) | |  |  |  |  |  |  |
|  | Never | 1 (Ref.) | 1 (Ref.) | 1 (Ref.) | 1 (Ref.) | 1 (Ref.) | 1 (Ref.) |
|  | < 1 | 0.92 (0.88, 0.96) | 0.90 (0.86, 0.94) | 0.93 (0.88, 0.97) | 0.90 (0.85, 0.94) | 0.95 (0.83, 1.09) | 0.94 (0.82, 1.08) |
|  | ≥ 1 | 0.89 (0.85, 0.94) | 0.87 (0.83, 0.92) | 0.89 (0.84, 0.94) | 0.86 (0.81, 0.91) | 0.97 (0.83, 1.12) | 0.94 (0.81, 1.10) |
| Duration of HRT (years) | |  |  |  |  |  |  |
|  | Never | 1 (Ref.) | 1 (Ref.) | 1 (Ref.) | 1 (Ref.) | 1 (Ref.) | 1 (Ref.) |
|  | < 2 | 0.87 (0.83, 0.92) | 0.85 (0.81, 0.89) | 0.89 (0.84, 0.94) | 0.85 (0.80, 0.90) | 0.83 (0.71, 0.97) | 0.83 (0.72, 0.97) |
|  | 2–5 | 0.83 (0.77, 0.90) | 0.81 (0.75, 0.88) | 0.86 (0.79, 0.94) | 0.82 (0.75, 0.89) | 0.81 (0.64, 1.02) | 0.82 (0.65, 1.04) |
|  | ≥ 5 | 0.78 (0.72, 0.84) | 0.76 (0.71, 0.83) | 0.79 (0.73, 0.87) | 0.76 (0.70, 0.83) | 0.59 (0.45, 0.77) | 0.60 (0.46, 0.78) |

Abbreviations: OC, oral contraceptives; HRT, hormone replacement therapy.

^*^Adjusted for age, body mass index, level of income, current smoking, drinking status, regular exercise, diabetes mellitus, hypertension, and dyslipidemia

**Supplementary Table 2. Hazard ratios and 95% confidence intervals of the duration of hormone therapy on the diagnosis of dementia including those who responded as “unknown” to the questions about hormone therapy**

|  |  | **All dementia** | | **Alzheimer’s disease** | | **Vascular dementia** | |
| --- | --- | --- | --- | --- | --- | --- | --- |
|  |  | **Crude** | **Adjusted^*^** | **Crude** | **Adjusted^*^** | **Crude** | **Adjusted^*^** |
|  |  | **Hazard ratio (95% Confidence interval)** | | | | | |
| Duration of OC use (years) | |  |  |  |  |  |  |
|  | Never | 1 (Ref.) | 1 (Ref.) | 1 (Ref.) | 1 (Ref.) | 1 (Ref.) | 1 (Ref.) |
|  | < 1 | 0.71 (0.68, 0.74) | 0.92 (0.88, 0.97) | 0.70 (0.67, 0.74) | 0.92 (0.88, 0.97) | 0.76 (0.66, 0.87) | 0.97 (0.85, 1.11) |
|  | ≥ 1 | 0.79 (0.75, 0.83) | 0.92 (0.88, 0.97) | 0.77 (0.73, 0.82) | 0.91 (0.86, 0.96) | 0.89 (0.77, 1.03) | 1.02 (0.88, 1.18) |
|  | Unknown | 0.97 (0.92, 1.02) | 0.99 (0.94, 1.04) | 0.97 (0.91, 1.02) | 0.99 (0.93, 1.05) | 0.97 (0.83, 1.13) | 0.99 (0.84, 1.18) |
| Duration of HRT (years) | |  |  |  |  |  |  |
|  | Never | 1 (Ref.) | 1 (Ref.) | 1 (Ref.) | 1 (Ref.) | 1 (Ref.) | 1 (Ref.) |
|  | < 2 | 0.43 (0.41, 0.45) | 0.85 (0.80, 0.89) | 0.42 (0.40, 0.45) | 0.85 (0.80, 0.90) | 0.44 (0.38, 0.51) | 0.83 (0.71, 0.96) |
|  | 2–5 | 0.39 (0.36, 0.42) | 0.81 (0.75, 0.87) | 0.39 (0.36, 0.43) | 0.81 (0.75, 0.89) | 0.42 (0.33, 0.53) | 0.81 (0.64, 1.02) |
|  | ≥ 5 | 0.49 (0.46, 0.53) | 0.81 (0.75, 0.87) | 0.49 (0.45, 0.53) | 0.81 (0.74, 0.88) | 0.42 (0.32, 0.53) | 0.65 (0.50, 0.84) |
|  | Unknown | 0.95 (0.90, 1.01) | 1.02 (0.96, 1.09) | 0.96 (0.90, 1.03) | 1.04 (0.97, 1.11) | 0.92 (0.76, 1.10) | 0.98 (0.80, 1.19) |

Abbreviations: OC, oral contraceptives; HRT, hormone replacement therapy.

^*^Adjusted for age, body mass index, level of income, current smoking, drinking status, regular exercise, diabetes mellitus, hypertension, and dyslipidemia

**Supplementary Table 3. Hazard ratios and 95% confidence intervals of the duration of hormone therapy on the diagnosis of dementia excluding those who were diagnosed with dementia 3 years after the diagnosis of depression**

|  |  | **All dementia** | | **Alzheimer’s disease** | | **Vascular dementia** | |
| --- | --- | --- | --- | --- | --- | --- | --- |
|  |  | **Crude** | **Adjusted^*^** | **Crude** | **Adjusted^*^** | **Crude** | **Adjusted^*^** |
|  |  | **Hazard ratio (95% Confidence interval)** | | | | | |
| Duration of OC use (years) | |  |  |  |  |  |  |
|  | Never | 1 (Ref.) | 1 (Ref.) | 1 (Ref.) | 1 (Ref.) | 1 (Ref.) | 1 (Ref.) |
|  | < 1 | 0.72 (0.69, 0.75) | 0.92 (0.88, 0.97) | 0.72 (0.68, 0.76) | 0.93 (0.88, 0.98) | 0.71 (0.60, 0.83) | 0.88 (0.75, 1.02) |
|  | ≥ 1 | 0.80 (0.76, 0.84) | 0.91 (0.86, 0.96) | 0.78 (0.74, 0.83) | 0.90 (0.85, 0.96) | 0.84 (0.71, 1.00) | 0.93 (0.78, 1.10) |
| Duration of HRT (years) | |  |  |  |  |  |  |
|  | Never | 1 (Ref.) | 1 (Ref.) | 1 (Ref.) | 1 (Ref.) | 1 (Ref.) | 1 (Ref.) |
|  | < 2 | 0.44 (0.42, 0.46) | 0.84 (0.80, 0.89) | 0.43 (0.40, 0.45) | 0.83 (0.78, 0.88) | 0.47 (0.40, 0.55) | 0.85 (0.72, 1.00) |
|  | 2–5 | 0.39 (0.36, 0.43) | 0.78 (0.72, 0.85) | 0.39 (0.36, 0.43) | 0.79 (0.72, 0.87) | 0.40 (0.31, 0.53) | 0.76 (0.58, 0.99) |
|  | ≥ 5 | 0.49 (0.45, 0.53) | 0.78 (0.71, 0.84) | 0.49 (0.45, 0.54) | 0.78 (0.71, 0.86) | 0.37 (0.27, 0.50) | 0.56 (0.41, 0.77) |

Abbreviations: OC, oral contraceptives; HRT, hormone replacement therapy.

^*^Adjusted for age, body mass index, level of income, current smoking, drinking status, regular exercise, diabetes mellitus, hypertension, and dyslipidemia

**Supplementary Table 4. Hazard ratios and 95% confidence intervals of the duration of hormone therapy on the diagnosis of dementia adding age at menopause as an adjusting variable**

|  |  | **All dementia** | | **Alzheimer’s disease** | | **Vascular dementia** | |
| --- | --- | --- | --- | --- | --- | --- | --- |
|  |  | **Crude** | **Adjusted^*^** | **Crude** | **Adjusted^*^** | **Crude** | **Adjusted^*^** |
|  |  | **Hazard ratio (95% Confidence interval)** | | | | | |
| Duration of OC use (years) | |  |  |  |  |  |  |
|  | Never | 1 (Ref.) | 1(Ref.) | 1 (Ref.) | 1(Ref.) | 1 (Ref.) | 1(Ref.) |
|  | < 1 | 0.71 (0.68, 0.74) | 0.92 (0.88, 0.96) | 0.71 (0.67, 0.74) | 0.92 (0.88, 0.97) | 0.76 (0.66, 0.87) | 0.96 (0.84, 1.10) |
|  | ≥ 1 | 0.78 (0.74, 0.82) | 0.91 (0.86, 0.95) | 0.77 (0.73, 0.81) | 0.90 (0.85, 0.95) | 0.87 (0.75, 1.01) | 0.98 (0.84, 1.14) |
| Duration of HRT (years) | |  |  |  |  |  |  |
|  | Never | 1 (Ref.) | 1 (Ref.) | 1 (Ref.) | 1 (Ref.) | 1 (Ref.) | 1 (Ref.) |
|  | < 2 | 0.43 (0.41, 0.45) | 0.84 (0.80, 0.88) | 0.43 (0.40, 0.45) | 0.84 (0.80, 0.89) | 0.44 (0.38, 0.51) | 0.83 (0.71, 0.96) |
|  | 2–5 | 0.40 (0.37, 0.43) | 0.81 (0.75, 0.87) | 0.39 (0.36, 0.43) | 0.81 (0.74, 0.89) | 0.42 (0.33, 0.53) | 0.81 (0.64, 1.03) |
|  | ≥ 5 | 0.48 (0.45, 0.52) | 0.78 (0.72, 0.84) | 0.48 (0.44, 0.52) | 0.78 (0.72, 0.85) | 0.39 (0.30, 0.51) | 0.61 (0.47, 0.80) |

Abbreviations: OC, oral contraceptives; HRT, hormone replacement therapy.

^*^Adjusted for age, body mass index, level of income, current smoking, drinking status, regular exercise, diabetes mellitus, hypertension, dyslipidemia, and age at menopause
